# *Streptococcus mutans* Proteases Degrade Dentinal Collagen

**DOI:** 10.3390/dj10120223

**Published:** 2022-11-28

**Authors:** Bo Huang, Cameron A. Stewart, Christopher A. McCulloch, J. Paul Santerre, Dennis G. Cvitkovitch, Yoav Finer

**Affiliations:** 1 Faculty of Dentistry, University of Toronto, Toronto, ON M5G 1G6, Canada; 2 Institute of Biomedical Engineering, University of Toronto, Toronto, ON M5S 3G9, Canada

**Keywords:** proteolytic activity, dentinal collagen, degradation, cariogenic bacterium

## Abstract

Here, we explored the role of *S. mutans*’s whole cell and discrete fractions in the degradation of type I collagen and dentinal collagen. Type I collagen gels and human demineralized dentin slabs (DS) were incubated in media alone or with one of the following: overnight (O/N) or newly inoculated (NEW) cultures of *S. mutans* UA159; intracellular proteins, supernatant or bacterial membranes of O/N cultures. Media from all groups were analyzed for protease-mediated release of the collagen-specific imino acid hydroxyproline. Images of type I collagen and DS were analyzed, respectively. Type I collagen degradation was highest for the supernatant (*p* < 0.05) fractions, followed by intracellular components and O/N cultures. Collagen degradation for DS samples was highest for O/N samples, followed by supernatant, and intracellular components (*p* < 0.05). There was lower detectable degradation for both type I collagen and DS from NEW culture samples (*p* < 0.05), and there was no type I collagen or DS degradation detected for bacterial membrane samples. Structural changes to type I collagen gel and dentinal collagen were observed, respectively, following incubation with *S. mutans* cultures (O/N and NEW), intracellular components, and supernatant. This study demonstrates that intracellular and extracellular proteolytic activities from *S. mutans* enable this cariogenic bacterium to degrade type I and dentinal collagen in a growth-phase dependent manner, potentially contributing to the progression of dental caries.

## 1. Introduction

Dental caries or tooth decay is defined as dissolution of tooth inorganic mineral components, primarily hydroxyapatite, by acid end-products from cariogenic bacteria, such as *Streptococcus mutans* (*S. mutans*), which results in the exposure of the organic dentinal collagen, primarily type I collagen. It has been suggested that dentinal collagen degradation due to proteolytic activity follows demineralization and complements the initial degradative effect of bacterial acids on the dentinal mineral structure, contributing to caries progression [1].

Potential sources of proteolytic activities that could contribute to dentinal collagen degradation are endogenous dentinal proteases [2], the oral microflora [3,4], and neutrophils [5]. Previous studies focused mainly on the role of degradative activities from endogenous matrix metalloproteinases (MMPs) in the hydrolysis of dentinal collagen [1,2]. Studies have shown that activated MMPs are involved in caries formation [6] and collapse of adhesive interface [2,7,8]. However, the contribution of endogenous MMPs to dentin degradation is controversial due to their limited abundance within dentin and limited enzymatic activity compared with bacteria and neutrophils [9]. In addition, the enzymatic activity of dentinal MMPs is not defined [10].

Bacterial proteolytic activities have been investigated because of their roles in bacterial invasion and tissue destruction in human diseases [11,12]. Human isolates of *S. mutans* have been shown to cause extensive bone loss and the breakdown of the periodontal ligament in gnotobiotic rats [13], which is suggested to be related to the bacteria’s proteolytic activity as demonstrated by their ability to degrade rat tail tendons [12]. The genes that encode *S. mutans* collagenolytic proteases have been identified by RNA sequencing and bio-informative methods from root caries [14]. Further, a MMP-like protein has been identified from *Streptococcus mitis* (*S. mitis*) [15]. However, none of these studies have directly linked the specific proteolytic activity of *S. mutans* to human dentinal collagen degradation, and there are no data related to where such bacterial collagenolytic/gelatinolytic protease activities reside: intracellular, excreted extracellularly, or in the bacterial membrane.

Hence, further exploration of the proteolytic activity of the cariogenic bacteria *S. mutans* on dentinal degradation and its potential impact on the pathogenesis of primary and secondary caries is warranted. The aim of the current study was to investigate the role of whole cell *S. mutans’s* and discrete fractions of this bacterium in the degradation of type I collagen and human dentin-derived collagen. The hypothesis is that *S. mutans* expresses proteolytic activities, located in defined cell extract fractions of the bacterium, that degrade purified type I collagen and dentinal collagen.

## 2. Materials and Methods

### 2.1. Type I Collagen Degradation by S. mutans UA159 and Its Discrete Fractions

Type I collagen gels were formed as previously described with modifications [16]. Type I rat tail collagen (3 mg/mL) (Gibco™ Collagen I, Fisher Scientific, Ottawa, ON, Canada) was mixed with 10× phosphate-buffered saline (PBS) to a final concentration of 1 mg/mL. The pH was adjusted to 7.4 by 1 N NaOH. Then, 150 μL of solution was dispensed into glass bottom dishes (50 mm, uncoated, MatTek Corporation, MA, USA), and incubated at 37 °C in a humidified incubator for 2 h until a firm gel was formed.

Overnight (O/N) cultures of *S. mutans* UA159 were prepared by inoculating ¼ Todd-Hewitt-Yeast extract (THYE, with 50 mM MOPS, pH 7.2) (Becton, Dickinson and Company, MD, USA) and incubated at 37 °C for 12 h. 1:100 fresh inoculated cultures (NEW) were prepared using ¼ THYE incubated at 37 °C for 4 h. Whole bacteria cells and supernatant (cell-free fraction) from O/N culture were separated by centrifugation (8000× *g*, 20 min, Hermle Z400K, Labnet International, Inc., Edison, NJ, USA) and collected. A portion of the cells were disrupted using an ultrasonic homogenizer (20 kHz, Branson Ultrasonics™ Sonifier™ SFX150 Cell Disruptor, Fisher Scientific, Danbury, CT, USA) and intracellular and bacterial membrane fractions of *S. mutans* were separated, concentrated, and collected (protocol in the Appendix A). Then, collagen gels (n = 3/group) were exposed to 200 μL of the following at 37 °C for 24 h:

(1) 125 CDU/mL *Clostridium histolyticum* (*C. histolyticum*) collagenase (0.2 mg) (positive control to validate the assay)
(2) ¼ THYE medium (negative control to exclude degradative effect from medium)
(3) Overnight (O/N) culture of *S. mutans* UA159 (OD600 = 0.8) in ¼ THYE
(4) 1:100 fresh inoculated culture (NEW) of *S. mutans* UA159 (concentrated to OD600 = 0.8) in ¼ THYE
(5) Intracellular protein fraction of lysed O/N *S. mutans* UA159 (see further details in the Appendix A)
(6) Supernatant (cell-free fraction) from O/N of *S. mutans* UA159 culture
(7) Bacterial membrane fraction of lysed O/N *S. mutans* UA159 (see further details in the Appendix A)

Medium from each dish was collected for hydroxyproline assay by ultra-performance liquid chromatography and mass spectrometry assay as described previously (UPLC-MS, ACQUITY LC system and Xevo G2-XS QToF, Waters, Mississauga, ON, Canada) [17]. Media was also used for calculation of the dry weight of cells and results were normalized by cell mass.

Collagen gels from each group were washed with PBS and kept humidified during imaging examination. The auto-fluorescence of collagen gels was acquired to visualize structural destruction using confocal laser scanning microscopy (CLSM; DMI6000 Inverted Microscope, Leica TCS SP8, Wetzlar, Germany, 488 nm Argon gas laser, Two Photo Multipliers Tube (PMT) detector, resolution 1024 × 1024 pixels) and an oil immersion objective (63x/1.40 HC PLAPO CS2) with excitation at 480 nm and emission at 490 nm [18].

### 2.2. Dentinal Collagen Degradation by S. mutans UA159 and Its Discrete Fractions

O/N cultures of *S. mutans* UA159, NEW cultures, and bacterial fractions (supernatant, intracellular components, and membranes) were prepared as described above. Dentin slabs (3 mm × 3 mm × 1 mm) were prepared from human molars (University of Toronto, Toronto, ON, Canada, Human Ethics Protocols #25793; 32320), demineralized in 10% phosphoric acid for 18 h, and incubated (n = 3/group) with 200 µL of the samples from the different degradation condition groups described above, at 37 °C for 2 weeks with replacement of O/N and NEW cultures every 24 h.

Media from each group were collected and analyzed for hydroxyproline content as described above [17]. Dentin samples were rinsed 3 times by PBS and gradually dehydrated with a series of ethanol solution (30%, 50%, 70%, 90% and 100%). Then, samples were freeze-dried and coated with gold (SC515SEM coating system; Polaron Equipment Ltd., Laughton, East Sussex, UK). Dentinal structural changes were visualized using scanning electron microscopy (SEM; FlexSEM 1000, Hitachi Co., Tokyo, Japan) [19]. CLSM was not performed on dentinal samples due to the masking effect by autofluorescence from compacted collagen structures.

### 2.3. Statistical Analysis

Background measurements from the negative control values (media only) were subtracted from the values of the experimental groups. One-way analyses of variance (ANOVA) and Scheffe’s multiple comparison tests (*p* < 0.05) were performed to validate differences in isolated hydroxyproline from type I collagen or dentin samples among experimental groups incubated with different cultures and bacterial fractions. Homogeneity of variance and normality were verified with Leven’s and Shapiro–Wilk tests, respectively (*p* < 0.05).

### 2.4. Verification of Dentinal Collagen Degradation by Intracellular Proteins of S. mutans Using SDS-PAGE and Mass Spectrometry

Equal amounts of intracellular proteins (75 μg) were collected from O/N cultures of *S. mutans* UA 159 as described above [20]. Demineralized dentin slabs were prepared as described above, then incubated at 37 °C for 2 weeks, with one of the following (n = 3/group):

(1) *C. histolyticum* collagenase (positive control)
(2) PBS (negative control)
(3) 75 μg of protein (experimental groups)

In addition, pure *C. histolyticum* collagenase and extracted intracellular proteins of *S. mutans* were incubated for 2 weeks without dentin samples as benchmark controls.

The media containing dentinal collagen degradation products from all groups were separated by gel electrophoresis. Collagen degradation products within the incubation media were verified by 15% sodium dodecyl sulfate-polyacrylamide gel electrophoresis (SDS-PAGE) as described previously [21]. The suspected collagen degradation fragments were present as bands on SDS-PAGE gels. The two most distinct bands of interest from experimental groups were collected, digested and analyzed by mass spectrometry as described previously [22] using LC-MS/MS (ThermoFisher LTQ, SPARC Biocentre at the Hospital for Sick Children, Toronto, ON, Canada). All MS/MS samples were analyzed using MS-Amanda Proteome Discoverer (Research Institute of Molecular Pathology, Vienna, Austria; version AmandaPeptideIdentifier in Proteome Discoverer 2.2.0.388).

## 3. Results

### 3.1. Type I Collagen Degradation by S. mutans UA159 and Its Discrete Fractions

**Figure 1:**
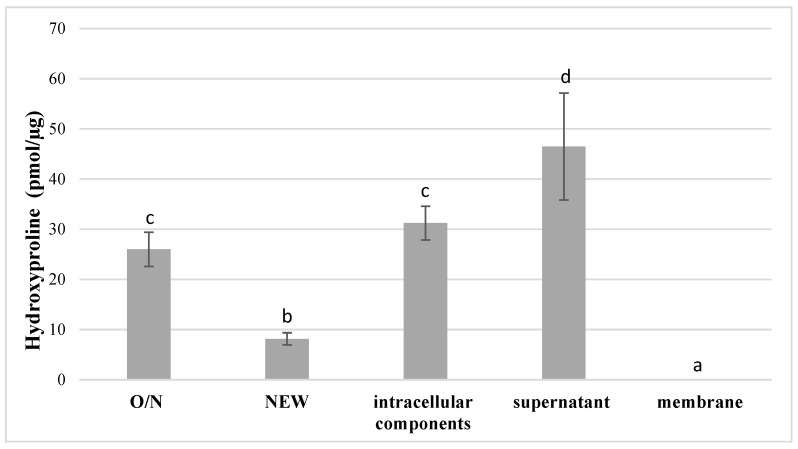
Hydroxyproline production after incubation of type I collagen with O/N and NEW *S. mutans* UA159 and its discrete fractions (n = 3; data are reported as mean ± standard errors). Different lowercase letters indicate statistically significant differences between the groups. Values with the same letters indicate non-significant differences (*p* > 0.05). O/N: overnight cultures; New: 1:100 fresh inoculated cultures.

Hydroxyproline release for type I collagen was normalized to bacterial weight and mass of collected components. The positive control (purified *C. histolyticum* collagenase) confirmed the specificity of the assay, and the negative control (media only) confirmed that there was no degradation activity associated with the medium itself (data not shown), and hence any measured degradation activity would be associated with elements of *S. mutans.* The supernatant of *S. mutans* growth media had the highest degradative activity towards type I collagen, producing 46.5 ± 10.7 pmol hydroxyproline per μg substrate (*p* < 0.05), followed by that of the intracellular component (31.2 ± 3.4 pmol/μg), which was not statistically different from O/N bacteria culture (26.0 ± 3.4 pmol/μg) (*p* > 0.05) (Figure 1). The lowest amount of hydroxyproline release (8.2 ± 1.2 pmol/μg) was recorded from newly inoculated *S. mutans* UA159 (NEW) (*p* < 0.05). There was no evidence of increased hydroxyproline release for the bacterial membrane samples, and therefore no activity towards Type I collagen gel (Figure 1).

**Figure 2:**
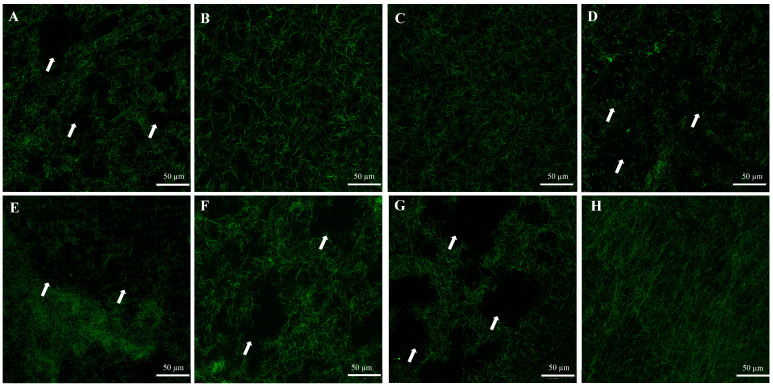
CLSM averaged images of autofluorescence of type I collagen gels incubated with (**A**) *C. histolyticum* collagenase (positive control), (**B**) PBS (negative control), (**C**) ¼ THYE medium (negative control), (**D**) O/N *S. mutans*, (**E**) NEW *S. mutans*, (**F**) intracellular components, (**G**) supernatant and (**H**) bacterial membrane. The arrows point out structural changes (loss of collagen fibrils).

CLSM averaged images of the autofluorescence of type I collagen gels from the negative controls showed well-established and intact collagen structures, while positive controls showed amorphous and disrupted collagen structures (Figure 2). The disrupted collagen structures were also observed in groups incubated with *S. mutans* (New and O/N), intracellular, and supernatant components (Figure 2).

### 3.2. Dentinal Collagen Degradation by S. mutans UA159 and Its Discrete Fractions

**Figure 3:**
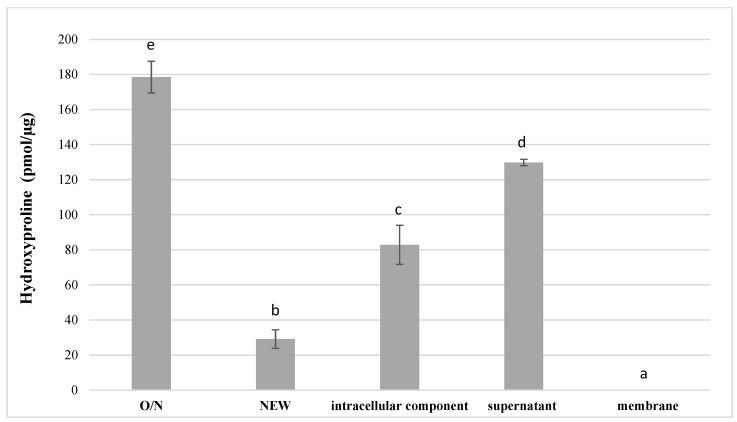
Hydroxyproline released into media after incubation of dentinal collagen slabs with O/N and NEW *S. mutans* UA159 and its discrete fractions (n = 3; data are reported as mean ± standard errors). Different lowercase letters indicate statistically significant differences between the groups. Values with the same letters indicate non-significant differences (*p* > 0.05).

The proteolytic activity of *S. mutans* UA159 and its discrete fractions towards dentinal collagen are depicted in Figure 3. The results were normalized to total mass of collected components. The medium alone, and the bacterial membrane fraction, yielded no activity towards dentinal collagen. O/N culture of *S. mutans* has the highest degradative activity towards dentinal collagen producing 178.5 ± 9.0 pmol/μg hydroxyproline (*p* < 0.05), followed by supernatant (129.8 ± 1.2 pmol/μg) and intracellular component (82.8 ± 11.2 pmol/μg) (*p* < 0.05). The lowest detectable amount of hydroxyproline (29.1 ± 5.3 pmol/μg) was released from dentin slabs incubated with newly inoculated *S. mutans* UA159 (*p* < 0.05).

**Figure 4:**
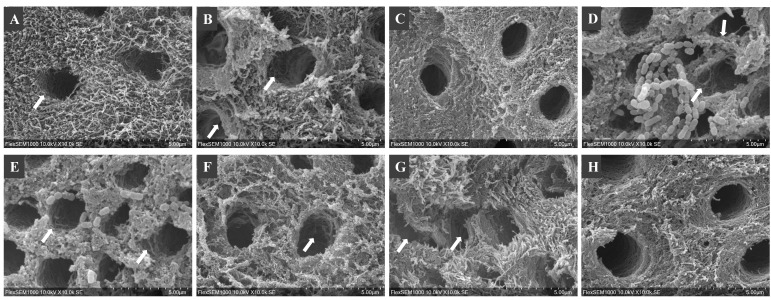
SEM images of dentinal collagen: (**A**) baseline of demineralized dentin (10% phosphoric acid for 18 h), incubated with (**B**) *C. histolyticum* collagenase (positive control), (**C**) ¼ THYE medium (negative control), (**D**) O/N *S. mutans*, (**E**) NEW *S. mutans*, (**F**) intracellular components, (**G**) supernatant and (**H**) bacterial membrane. The arrows point to significant structural disruption of dentinal tubules.

SEM images of demineralized dentin showed round, aligned and intact dentinal tubules and distinct dentinal collagen fibers, while the positive control showed enlarged and disrupted dentinal tubules (Figure 4). The accumulation of medium components and bacterial proteins deposited on the dentin surfaces over the long-term incubation masked some of the structural changes on the surfaces. However, the disrupted outline of the dentinal tubules can be observed in groups incubated with O/N cultures, intracellular, and supernatant components (Figure 4).

### 3.3. Verification of Dentinal Collagen Degradation by Intracellular Proteins of S. mutans Using SDS-PAGE and Mass Spectrometry

**Figure 5:**
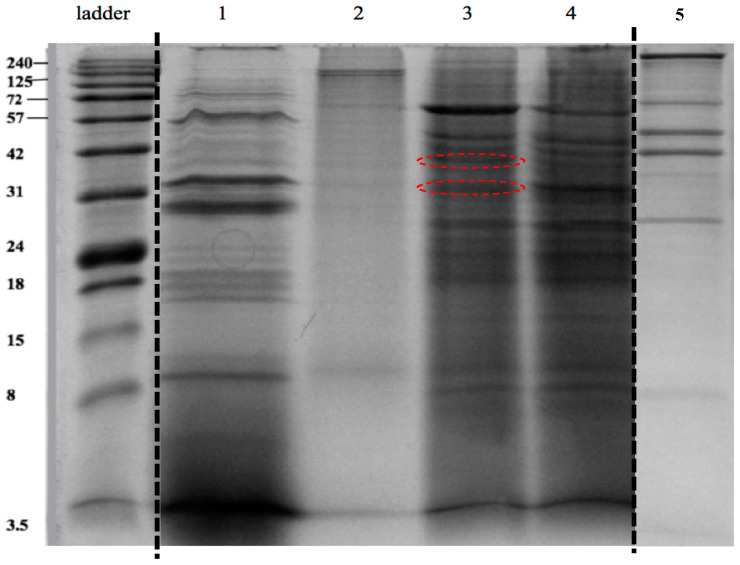
Identification of the dentin collagen degradation products following digestion with the extracted intracellular proteins of *S. mutans* UA159. Lane 1: positive control (dentin collagen samples incubated with *C. histolyticum* collagenase); lane 2: negative control (dentin collagen samples incubated in PBS); lane 3 and 4: experimental groups (dentin collagen samples incubated in intracellular proteins at 37 °C for 2 weeks); lane 5: intracellular protein extracted from *S. mutans* (baseline control). Extra groups were cut off from the same gel image between the ladder and Lane 1; Lane 5 (baseline control) was regrouped from another gel. Red circles indicate the most distinct bands of interest from experimental groups.

Fragments of dentinal collagen degraded by intracellular proteins are presented as peptide bands on the SDS-PAGE gel image (Figure 5). The bands in lane 1 represent collagen fragments that were the result of digestion by the *C. histolyticum* collagenase; in lane 2 (negative control), there are only two distinct bands with similar masses, around 120 KDa, representing typical α1- and α2-chains of type 1 collagen, and there is a smear spreading from 120 Kda to 8 Kda. In lanes 3 and 4, the bands represent *S. mutans* UA159 intracellular proteins and degraded dentinal collagen fragments. In lane 5, the bands represent the intracellular proteins extracted from *S. mutans* UA159. When comparing dentin specimens incubated with PBS (controls, lane 2) and extracted bacterial protein of *S. mutans* UA159 (lane 5), multiple extra bands were evident in lanes 3 and 4 (Figure 5). The numbers and distribution of peptide bands from groups incubated with *S. mutans* proteins (lane 3 and 4) were different compared with the group incubated with *C. histolyticum* collagenase (lane 1) (Figure 5).

**Figure 6:**
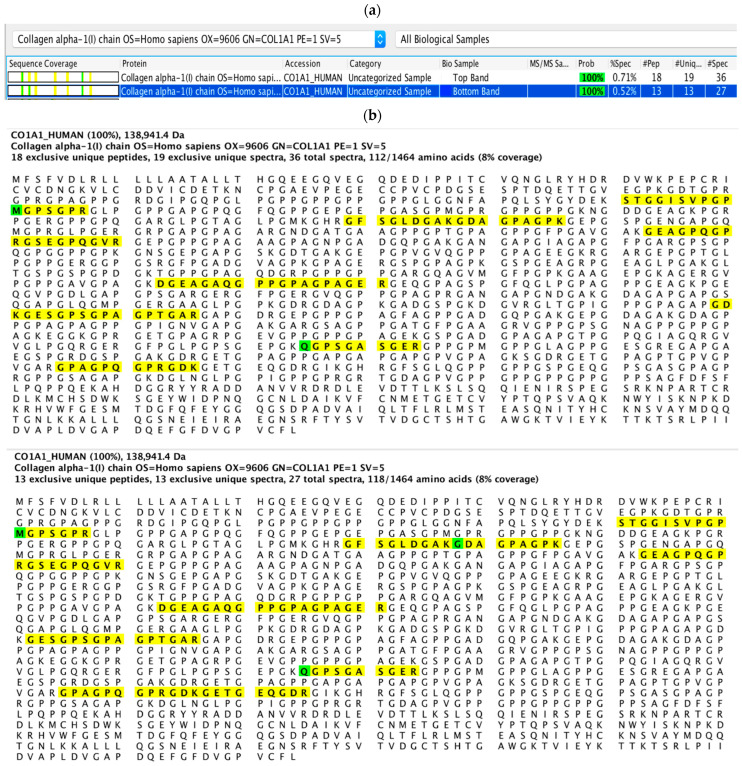
Identification of peptide sequence from dentinal collagen degradation by *S. mutans* UA159 intracellular proteins. The origin of degraded fragments was identified based on sequence homology against human type I collagen (**a**); peptide sequences were highlighted in sequences of human type I collagen α1 chain (**b**).

Two of the resultant fragments from collagen degradation by *S. mutans* proteins were identified as peptide fragments from α1 chain of human type I collagen based on sequence homology and specificity analysis (Figure 6a). These resultant peptide sequences were highlighted in the sequence of human type I collagen α1 chain (Figure 6b).

## 4. Discussion

Bacterial proteolytic activities have been previously characterized [3] and studied in the context of nutrient acquisition as a primary mechanism for bacterial survival [23,24]. In addition, these activities have been linked to direct and/or indirect host tissue destruction as virulence factors in several oral diseases [11]. From the many bacterial species that colonize and persist in the oral cavity, *S. mutans* is one of the few that have been consistently linked to caries formation, due in part to its ability to form biofilms, produce acid, and tolerate acidic conditions [25]. The strain used in the current investigation, *S. mutans* UA159 is a clinical isolate that degrade restorative materials and as such may contribute to marginal deterioration and secondary caries development [26]. The current investigation is the first to report on the proteolytic activity of *S. mutans* and its discrete fractions, and their ability to degrade type I collagen and more importantly, demineralized human dentin collagen. On the basis of these findings, we accept the hypothesis that *S. mutans,* a major pathogen involved in the pathogenesis of dental caries, expresses proteolytic activities located in defined cell extract fractions of the bacterium that degrades type I and dentinal collagen. This notion suggests new possibilities for these proteolytic activities to provide an alternative cariogenic mechanism for *S. mutans*.

The specificity and activity/efficiency of bacterial proteases vary towards different substrates [21]. Purified rat tail type I collagen is a relevant, reproducible and practical substrate to investigate the degradative activity of *S. mutans* towards dentinal collagen, since approximately 90% of the organic matrix in dentin is type I collagen [27]. Further, rat tail collagen has been used as substrate to investigate oral bacterial collagenolytic activity in previous studies [21,28,29,30]. The amino acid sequence of type I collagen comprises 33% glycine and also contains hydroxyproline and proline, which are important for the structure and function of collagen. Although bacterial collagenolytic enzymes cleave collagen at different sites and generate multiple degradation fragments, the release of hydroxyproline is a sensitive and reliable measure to estimate collagen degradation [4,31]. The results of the current investigation showed an important increase of hydroxyproline release from purified type I collagen in the presence of overnight and newly inoculated *S. mutans* cultures. As there was no detectable hydroxyproline release from media alone, we suggest that the bacterium is the sole source of this protease activity and can degrade type I collagen. This finding was supported qualitatively by CLSM, which also demonstrated the structural destruction of collagen gels after incubation with *S. mutans* cultures.

In the current investigation, the higher degradation of type I collagen by O/N *S. mutans* cultures when compared with NEW cultures suggests a growth-phase dependency for the degradative capacity of the bacterium. This finding may be explained by the autolysis of *S. mutans* in its later growth stage, a condition that facilitates cell wall turnover, cell division, assembly of secretion systems, resuscitation of dormant cells, and micro fratricide [32]. As a result, more intracellular enzymes are released into incubation medium, contributing to enhanced collagen degradation. The increased collagen degradative activity for overnight cultures could also be explained by the increased production of selective proteases in the late growth stage of *S. mutans,* which is part of bacterial adaptation strategies, in which some oral pathogens may digest host tissue such as collagen in order to release amino acids as a nutrient source [33].

When compared to rat tail type I collagen, human dentin collagen has a more complex structure at different hierarchical levels [34]. Cross-linked structures indicate the presence of collagen molecules that are more resistant to enzymatic degradation than collagen molecules found in other tissues [34,35]. Therefore, further experiments were carried out to verify the proteolytic activity of *S. mutans* towards demineralized human dentinal collagen. There was no hydroxyproline release from dentin or dentinal structural changes in samples treated with medium only in which dentinal MMPs were the only possible source of proteolytic activity. This finding supports the hypothesis that endogenous MMPs may exert relatively low effects on dentinal collagen degradation [36] due to the limited amount of MMPs in dentine and their inactive form [37]. In contrast, the marked increase of hydroxyproline release in samples treated with overnight (O/N) or fresh-inoculated (NEW) *S. mutans* confirmed the ability of these fractions to degrade demineralized human dentin, corroborating the results from rat tail type I collagen. This observation was also supported by the SEM analysis showed dentinal tubule structural disruption of these groups. In addition, the higher dentinal and type I collagen degradation by O/N *S. mutans* cultures than NEW cultures confirms that the degradative capacity of the bacterium is growth-phase dependent. The amount of hydroxyproline released from demineralized dentin was several-fold higher than that from type I collagen, which could be explained by the longer incubation times that were used with the dentin samples (14 days vs. 1-day). This finding suggests that these degradative proteases are stable to maintain their activity throughout extended incubation times. In addition, unlike the type I collagen degradation study, the O/N *S. mutans* had the highest hydroxyproline release for the dentinal collagen samples, which could be a result of the combined effects of growth-dependent selective enzymes production and increased release of intracellular enzymes by bacterial autolysis caused by the prolonged incubation and the accumulation of these enzymes.

To locate the protease activity responsible more precisely for type I collagen and dentinal collagen degradation, discrete bacterial fractions and the incubation media were investigated. Both intracellular components and supernatants were capable of cleaving type I and dentinal collagen, releasing hydroxyproline and disrupting collagen structures. Supernatants from *S. mutans* O/N cultures showed higher degradative activity than intracellular components, suggesting that significant protease activity may be secreted or released extracellularly. This finding is supported by previous reports that most bacterial collagenases are extracellular proteins involved in bacterial invasion [38,39]. However, it cannot be assumed that the proteases were only secreted, since intracellular proteases may be released into the extracellular environment by *S. mutans* autolytic activity as mentioned above [40,41]. Autolyzed bacteria account for 30% to 40% of the population in *S. mutans* biofilm, and significantly contribute to extracellular/supernatant proteolytic activity [42]. In addition, the current results verified that an important proportion of these proteases are located intracellularly, a conjecture that is based on the high hydroxyproline release from type I collagen and dentin samples incubated with intracellular proteins of *S. mutans*.

Bacterial collagenolytic proteases have a broad range of specificity, but their substrates are hydrolyzed at various specific peptide bonds [43]. The main source of knowledge of bacterial collagenases is based on multiple studies on the enzymes produced by *C. histolyticum* [44]. In the current study, purified collagenase from *C. histolyticum* was used as a positive control to analyze the degradative effects and patterns of proteases from *S. mutans*. As a baseline, type I collagen released from demineralized dentin incubated with PBS buffer were collected and were detected on SDS-PAGE gel as two distinct bands, indicating typical α1- and α2 chains of type I collagen. The absence of αA chains, which are the characteristic 3/4-cleavage products generated by mammalian interstitial collagenases, reinforces the limited effect of dentinal endogenous MMPs on dentinal collagen degradation [45]. The smear presented on SDS-PAGE gels of samples of demineralized dentin incubated with PBS buffer suggest that non-collagenous proteins were released after the demineralization procedure or were denatured collagen fragments. When comparing dentin specimens incubated with PBS control or extracted bacterial protein of *S. mutans* UA159, the collagen fragments degraded by *S. mutans* proteases were presented as multiple bands on SDS-PAGE gel.

The different bands generated by *S. mutans* intracellular proteases and *C. histolyticum* collagenase indicate that the collagen cleavage sites generated by *S. mutans* proteases are different than those of *C. histolyticum*, and that different bacterial enzymes are likely responsible for the cleavage. The primary-structural analysis of two resultant collagen fragments derived from dentinal collagen upon digestion with *S. mutans* intracellular proteins confirmed the degraded peptides were from the α1 chain of type I collagen, suggesting that the enzymes might preferentially act on certain peptide sequences. Although not all of the possible cleavage sites in collagen were determined, several preferred amino acids were suggested as cleavage sites, including Lys, Gly, Ser and Arg, which have been reported for other bacterial collagenolytic proteases [39,46,47]. However, the proteases’ specificity cannot be definitively identified due to the combined effects of multiple proteases in the intracellular components. Therefore, specific collagenolytic/gelatinolytic proteases from *S. mutans* have been synthesized for more detailed investigation, to be reported on in a follow-up investigation.

Although the current definition of caries is still limited to demineralization of dental tissues due to the acid produced by sugar-fermenting microorganisms, the current investigation proposes that dentinal collagen may be degraded by extracellular and intracellular cariogenic bacterial proteases after demineralization, which further highlights the potential multifactorial contributions of *S. mutans* to primary and secondary caries formation and progression. Further characterization of the bacterium degradative activity and degradative mechanisms, and identification of specific proteases that are involved in this process, is needed.

## 5. Conclusions

The current investigation verified the proteolytic activity of a clinical isolate of *S. mutans*, a major pathogen involved in the pathogenesis of dental caries, and the ability of this bacterium in to degrade dentinal collagen in a growth-phase dependent manner. The initial analysis suggest that the bacterial proteases originate from both intra- and extracellular origins.

## Data Availability

Not applicable.

## Appendix A

***Intracellular components acquisition***

Intracellular component fractions were prepared as described previously [40,48] with modifications. *S. mutans* UA 159 were grown in THYE (pH 7.0) at 37 °C (OD_600_ = 0.8). The cells were harvested by centrifugation at 5000× *g* for 10 min and washed with PBS. Then, the bacterial cells were re-suspended in PBS and subjected to ultrasonic homogenizer (20 kHz, Branson Ultrasonics™ Sonifier™ SFX150 Cell Disruptor, Fisher Scientific) for 3 min with cooling in between. Intact cells were sedimented by centrifugation at 16,000× *g* for 5 min at 4 °C. The resultant intracellular components were released into the supernatant which was separated from the sedimented fraction, collected and stored at −80 °C until required.

***Membrane pellet acquisition***

For membrane preparation, *S. mutans* UA 159 were grown in THYE (pH 7.0) at 37 °C (OD_600_ = 0.8). Membrane-enriched protein fractions were prepared as described previously [49,50], with some modifications. The bacterial cells were harvested by centrifugation at 5000× *g* for 10 min and washed with PBS. The cells were re-suspended in buffer A (20% sucrose, 20 mM Tris [pH 7.0], 10 mm MgCl2), and then in order to reduce cell wall protein contamination, mutanolysin (1 mg; Sigma, St. Louis, MO, USA) and lysozyme (18 mg; Sigma, St. Louis, MO, USA) were added and the mixture was incubated at 37 °C with gentle agitation. Protoplast formation was monitored using Gram staining. Once cell wall digestion was complete, mixtures were centrifuged as described above. The protoplasts were washed once with buffer A, and each cell pellet was suspended in buffer B (10 mM Tris [pH 8.1], 50 mM MgCl2, 10 mM glucose). The protoplasts were lysed. Debris and unbroken protoplasts were removed by centrifugation at 6000× *g* for 10 min at 4 °C, and the supernatant was ultracentrifuged at 41,000× *g* for 45 min at 4 °C. The pellet was suspended in buffer C (10 mM Tris [pH 8.1], 50 mM NaCl, 20 mM MgCl2) at 4 °C and ultracentrifuged at 105,000× *g* for 45 min at 4 °C. The supernatant was decanted, and the membrane-enriched pellet was washed twice with buffer D (20 mM Tris [pH 7.2], 10 mM MgCl_2_) and stored at −80 °C until it was used.
